# Individual Assessment of Arteriosclerosis by Empiric Clinical Profiling

**DOI:** 10.1371/journal.pone.0001215

**Published:** 2007-11-28

**Authors:** Marcus Mutschelknauss, Marco Kummer, Juergen Muser, Steve B. Feinstein, Peter M. Meyer, Barbara C. Biedermann

**Affiliations:** 1 University Department of Medicine, Kantonsspital Bruderholz, Bruderholz, Switzerland; 2 Department of Research, University Hospital Basel, Basel, Switzerland; 3 Department of Preventive Medicine, Rush University Medical Center, Chicago, Illinois, United States of America; Baylor College of Medicine, United States of America

## Abstract

**Background:**

Arteriosclerosis is a common cause of chronic morbidity and mortality. Myocardial infarction, stroke or other cardiovascular events identify vulnerable patients who suffer from symptomatic arteriosclerosis. Biomarkers to identify vulnerable patients before cardiovascular events occur are warranted to improve care for affected individuals. We tested how accurately basic clinical data can describe and assess the activity of arteriosclerosis in the individual patient.

**Methodology/Principal Findings:**

269 in-patients who were treated for various conditions at the department of general medicine of an academic tertiary care center were included in a cross-sectional study. Personal history and clinical examination were obtained. When paraclinical tests were performed, the results were added to the dataset. The numerical variables in the clinical examination were statistically compared between patients with proven symptomatic arteriosclerosis (n = 100) and patients who had never experienced cardiovascular events in the past (n = 110). 25 variables were different between these two patient groups and contributed to the disease activity score. The percentile distribution of these variables defined the empiric clinical profile. Anthropometric data, signs of arterial, cardiac and renal disease, systemic inflammation and health economics formed the major categories of the empiric clinical profile that described an individual patient's disease activity. The area under the curve of the receiver operating curve for symptomatic arteriosclerosis was 0.891 (95% CI 0.799-0.983) for the novel disease activity score compared to 0.684 (95% CI 0.600-0.769) for the 10-year risk calculated according to the Framingham score. In patients suffering from symptomatic arteriosclerosis, the disease activity score deteriorated more rapidly after two years of follow-up (from 1.25 to 1.48, P = 0.005) compared to age- and sex-matched individuals free of cardiovascular events (from 1.09 to 1.19, P = 0.125).

**Conclusions/Significance:**

Empiric clinical profiling and the disease activity score that are based on accessible, available and affordable clinical data are valid markers for symptomatic arteriosclerosis.

## Introduction

Arteriosclerosis is a common, chronic and progressive disorder of the large elastic and muscular arteries. It is complicated by cardiovascular events such as myocardial infarction, stroke or peripheral arterial occlusive disease [1]. Nearly 10% of patients in the primary care setting suffer from symptomatic arteriosclerosis, i.e. arteriosclerosis complicated by cardiovascular events [2]. It is the leading cause of death in developed countries and its prevalence is supposed to rise globally due to the worldwide increase of diabetes and obesity [3]–[5]. The annual event rate for patients with symptomatic arteriosclerosis is 4% [6]. Current preventive management of this chronic disease is based on risk assessment [7], [8] and treatment of risk factors such as arterial hypertension, smoking, diabetes or hypercholesterolemia. However, conditions that affect risk, particularly cardiovascular risk, are variable with time and geographic environment [9]. Therefore, contemporary and regional patient cohorts are needed for the timely adjustment of reference values. Furthermore, a test to determine the vulnerability to develop arteriosclerosis [10], [11] and eventually cardiovascular events would clearly improve the accuracy of risk prediction in the individual patient. The quest for biomarkers that are suitable to diagnose arteriosclerosis in its subclinical, asymptomatic stage is ongoing [12], [13]. But neither laboratory tests nor modern imaging modalities have substantially forwarded the field so far [14]–[19]. In addition, non-invasive coronary CT angiography and other diagnostic imaging modalities are currently criticized for extraordinary high radiation exposure leading to a significant increase in cancer [20], [21]. In order to address some of these issues, we used comprehensive clinical bedside examination and applied the rules of differential display for data analysis to determine the phenotype of patients with symptomatic arteriosclerosis or vulnerable patients in our hospital. We wished to test whether clinical findings, i.e. history taking, physical examination and a few additional, common and affordable bedside procedures would accurately describe the activity of arteriosclerosis in the individual patient. We found that this approach is at least as efficient as conventional risk assessment and novel biomarkers [22] for the detection of vulnerable patients.

## Methods

### Objectives

We tested to what extent basic, available and affordable clinical data obtained from the patient's history, physical examination and paraclinical tests can be used to assess accurately the phenotype and disease activity of patients with symptomatic arteriosclerosis.

### Participating Patients

Between September 2003 and March 2005, all 718 in-patients who were treated for any reason at a ward of the department of medicine were screened for exclusion criteria to participate in this study. Exclusion criteria were either inability to give informed consent or terminal illness. 431 patients without exclusion criteria were asked to participate. 162/431 (38%) patients refused and 269/431 (62%) patients gave written informed consent. These patients were grouped in three categories based on the clinical history: group 1–no cardiovascular events in the past; group 2–cardiovascular events in the past which define symptomatic arteriosclerosis; group 3–symptoms compatible with symptomatic arteriosclerosis, but clinical evidence to prove it was lacking. For the data-based clinical disease profile, patients without cardiovascular events (group 1) and patients with proven, symptomatic arteriosclerosis (group 2) were compared (Table 1). Cardiovascular events which defined symptomatic arteriosclerosis in this patient cohort were a) *for coronary heart disease:* myocardial infarction, angina pectoris with signs of myocardial ischemia, history of coronary bypass surgery or other revascularization procedures, b) *for cerebrovascular disease:* ischemic stroke, history of carotid surgery, c) *for peripheral arterial occlusive disease*: ankle brachial index<0.9 [23] and symptoms of claudicatio intermittens, significant stenosis of arteries and symptoms of claudicatio, history of peripheral bypass surgery or other revascularization procedure, d) *for aortic arteriosclerosis*: symptomatic aortic aneurysm, diameter of infrarenal aorta >3 cm [24], aortic surgery for arteriosclerosis and e) *for arteriosclerosis of the kidney*: renal artery stenosis, impaired renal function [25] with normal urine analysis, history of renal artery revascularization procedures. Male sex, arterial hypertension, diabetes mellitus, dyslipidemia, smoking and a positive family history for cardiovascular disease were six conventional cardiovascular risk factors which were assessed based on the clinical history [26].

**Table 1:** Patient characteristics

	No cardiovascular events (n = 110)	Symptomatic arteriosclerosis (n = 100)	P-values #
**Cardiovascular risk factors n (%)**			
*Male sex*	51 (46.4)	57 (57)	0.095
* Age (years)*	56.00	72.00	<0.001
* Body mass index (kg/m^2^)*	25.6	26.40	0.085
*Arterial hypertension*	40 (36.4)	67 (67)	<0.001
* Diabetes mellitus*	12 (10.9)	30 (30)	0.005
* Dyslipidemia*	9 (8.2)	50 (50)	<0.001
* Smoking*	57 (51.8)	61 (61)	0.017
* Family history of cardiovascular disease*	48 (43.6)	61 (61)	0.038
**Drugs at examination n (%)**			
*Antiplatetelet drugs*	7 (6.4)	69 (69)	<0.001
* Anticoagulants*	27 (24.6)	36 (36)	0.07
* Nitrates*	1 (0.9)	18 (18)	<0.001
* Betablockers*	19 (17.3)	60 (60)	<0.001
* Diuretics*	17 (15.5)	48 (48)	<0.001
*ACE inhibitors*	14 (12.7)	56 (56)	<0.001
* Angiotensin II receptor blockers*	8 (7.3)	11 (11)	0.347
* Ca^2+^ channel blockers*	7 (6.4)	26 (26)	<0.001
* Oral glucose-lowering agents*	9 (8.2)	14 (14)	0.178
* Insulin*	5 (4.6)	21 (21)	<0.001
* Statins*	14 (12.7)	68 (68)	<0.001
* Other drugs*	5 (4.6)	8 (8)	0.299
**Cardiovascular events defining symptomatic arteriosclerosis %**			
* Coronary heart disease*	*-*	60	
Myocardial infarction	-	49	
Significant stenosis of coronary arteries (angiographic findings)	-	23	
Angina pectoris with signs of myocardial ischemia (e.g. exercise testing)	-	9	
History of revascularization	-	14	
* Cerebrovascular disease*	*-*	26	
Ischemic stroke	-	26	
* Peripheral arterial occlusive disease*	*-*	27	
Ankle-brachial-index<0.9 and symptoms of claudicatio intermittens	-	14	
Angiographically proven and symptoms of claudicatio intermittens	-	5	
History of revascularization	-	17	
* Arteriosclerosis of the aorta*	*-*	7	
* Arteriosclerosis of the kidney*	*-*	11	
**Number of organs affected by cardiovascular events**			
1	-	72	
2	-	26	
≥3	-	2	

# The two patient groups were compared using the Mann-Whitney-U-Test (for numerical data) or the χ^2^-test (for non-numerical data)

### Ethics

The study protocol was approved by the independent ethical review board. Patients who fulfilled the inclusion criteria and were willing to participate gave written informed consent.

### Comprehensive clinical assessment

All participants were subject to a standardized interview (H, history) and examined in a standardized clinical examination (C). Body weight and size, waist and hip circumference, blood pressure and heart rate were measured on both arms in the standing position first. Thereafter, the examination was continued in the supine position. Blood pressure and heart rate measured in supine position were usually obtained at the end of the examination, together with the determination of the ankle brachial index (ABI) that was assessed using bedside doppler ultrasound (Dopplex 5 MHz, HNE Healthcare GmbH, Hilden, Germany) [23]. Patients with incompressible leg arteries had an ABI of more than 1.5. These excessively high indexes that were found in 13 patients were excluded from the dataset. The patient's record served as a source for additional information such as laboratory tests (L), X-rays (X), electrocardiogram (E), stress test or echocardiogram. No additional laboratory tests were performed except for those requested by the treating physicians. The full clinical assessment was entered into an electronic data base and included 75 numeric variables (see Table S1) that were selected for further analysis. 14 (19%) were obtained from the interview, 19 (25%) from the clinical examination, 33 (44%) from the laboratory tests and 9 (12%) from x-ray, electrocardiogram, stress test or echocardiogram. For 15 of these 75 parameters, the dataset was incomplete, i.e. information from less than 75% of the patients was available (Table S1).

### Empiric clinical disease profiling and disease activity score

**Figure 1:**
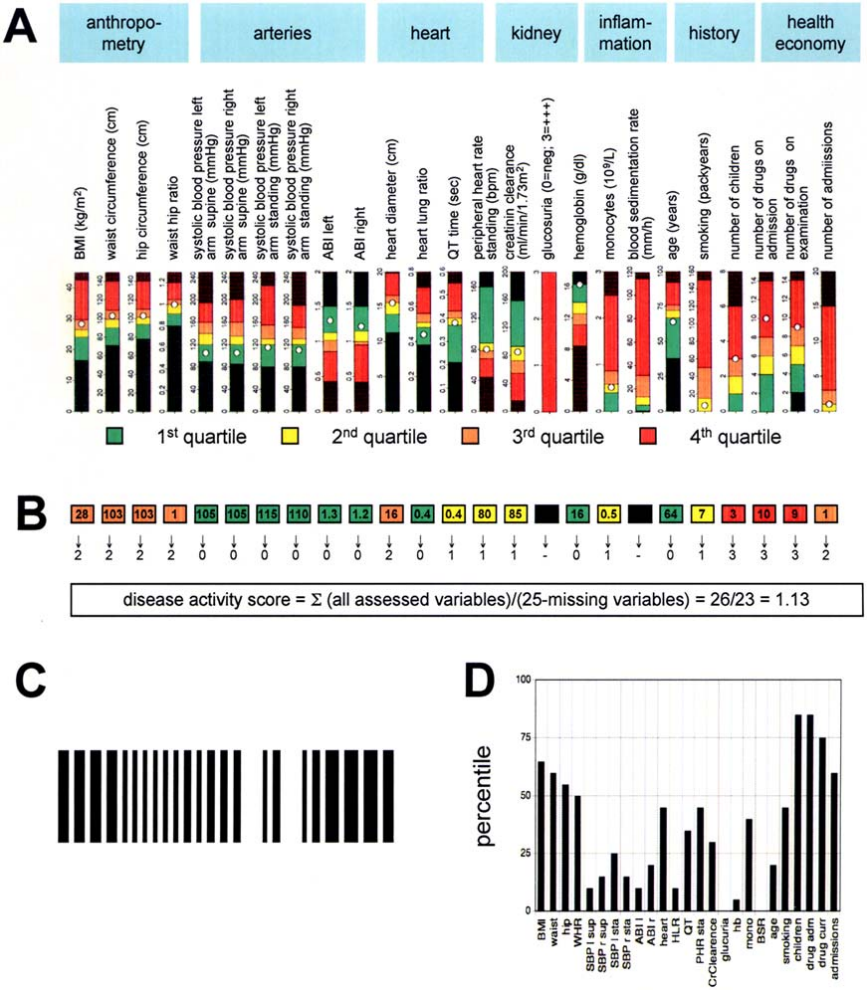
Empiric clinical profiles of arteriosclerosis and disease activity score. A. Reference range of data obtained from each of the 25 significantly different variables in symptomatic patients. An individual patient's data (represented as white dots) are superposed onto the color-coded quartile distribution. B. The same individual patient's color-coded empiric clinical disease profile of arteriosclerosis. The color-coded profile can be transformed into numbers: green = 0, yellow = 1, orange = 2 and red = 3. The arithmetic mean of these numbers is equivalent to the disease activity score. C. The color- or number-coded profiles can be transformed into a barcode. D. The individual patient's quantitative percentile profile.

The 60 nearly complete variables were compared between patients without cardiovascular events in the past (group 1) and patients with proven symptomatic arteriosclerosis (group 2) using the Mann Whitney U test (Table S1). For 25 variables (42%), the P-value was below 0.1 and these parameters were selected to be part of the data-based, empiric clinical disease profile (Table 2). For both groups, the percentile distribution of the data was calculated and the quartile ranges are shown (Table 2). The group of patients with the disease, i.e. with proven symptomatic arteriosclerosis (group 2, n = 100 patients) defined the empiric clinical disease profile. The quartile range served for color coding the patient's individual data (Table 2 and Figure 1A). For most of the numerical variables, patients with symptomatic arteriosclerosis had higher median values than the patients without cardiovascular events. Therefore, the lowest quartile was assigned light green, the 2^nd^ quartile yellow, the 3^rd^ quartile orange and the 4^th^ quartile red. Values below the minimal value of the data-set were coded as dark green and values above the maximal value were coded as dark red. Exceptions to this rule were the ankle brachial indexes, the peripheral heart rate at standing position, the creatinine clearance and the hemoglobin concentration. For these 5 variables, the patients with symptomatic arteriosclerosis had lower median values compared to the asymptomatic patients, and therefore colorcoding followed the opposite rule: the highest quartile range was assigned light green, the 3rd yellow, the 2^nd^ orange and the lowest quartile range red (Figure 1A). This mathematical transformation allows standardized representation of clinical data in various formats. For example, an individual patient's empiric clinical profile can be intuitively determined when his personal data are placed onto the color-coded reference range (Figure 1B). Alternatively, a disease activity score can be calculated as follows: for the light and dark green color, a variable score point of 0, for the yellow color a score point of 1, for the orange color a point of 2 and for the red and dark red color a point of 3 can be given (Figure 1B). The individual disease activity score can then be calculated as the average variable score point. The variable score points can also be transferred into machine-readable bar-codes (Figure 1C) that are useful for any digital processing and large-scale management of clinical data such as during epidemiologic studies, e-health applications or comparative assessment of health care quality. Finally, a more precise and quantitative clinical profile can be derived using percentile distributions with infinite resolution as shown in Figure 1D.

**Table 2:** The numerical data selected for the data-based clinical disease profile and the disease activity score

Parameter	Method §	No cardiovascular events (n = 110)					Symptomatic atherosclerosis (n = 100)					P-value #
		Maximum value	75. percentile	*Median*	25. Percentile	Minimum value	Maximum value	75. percentile	*Median*	25. percentile	Minimum value	
Body mass index (kg/m^2^)	C	40.1	27.75	**25.6**	23.25	14.5	42.40	29.73	**26.4**	24.07	16.59	0.085
Waist circumference (cm)	C	120.0	101.0	**95.0**	82.0	56.0	140.0	109.0	**99.5**	90.0	71.0	0.006
Hip circumference (cm)	C	126.0	105.0	**100.0**	91.0	71.0	124.0	110.0	**101.5**	94.0	78.0	0.078
Waist hip ratio	C	1.20	1.00	**0.94**	0.88	0.75	1.20	1.04	**1.00**	0.91	0.78	0.006
Systolic blood pressure left arm supine (mmHg)	C	180.0	140.0	**130.0**	110.0	85.0	195.0	160.0	**140.0**	120.0	90.0	0.003
Systolic blood pressure right arm supine (mmHg)	C	190.0	140.0	**130.0**	110.0	90.0	200.0	160.0	**135.0**	120.0	85.0	0.005
Systolic blood pressure left arm standing (mmHg)	C	180.0	140.0	**120.0**	100.0	80.0	225.0	155.0	**130.0**	120.0	80.0	0.001
Systolic blood pressure right arm standing (mmHg)	C	200.0	140.0	**120.0**	110.0	80.0	190.0	150.0	**130.0**	120.0	80.0	0.007
Ankle brachial index left *	C	1.45	1.21	**1.15**	1.07	0.70	1.50	1.13	**1.02**	0.86	0.43	<0.001
Ankle brachial index right *	C	1.46	1.24	**1.14**	1.06	0.76	1.50	1.15	**1.00**	0.95	0.42	<0.001
Heart diameter (cm)	X	17.7	14.9	**13.5**	12.5	9.40	19.8	16.6	**15.6**	13.9	11.3	<0.001
Heart lung ratio	X	0.61	0.49	**0.47**	0.43	0.35	0.71	0.56	**0.51**	0.48	0.38	<0.001
QT time (sec)	E	0.50	0.40	**0.37**	0.35	0.27	0.55	0.43	**0.40**	0.37	0.21	<0.001
Peripheral heart rate standing (bpm)	C	120.0	92.0	**80.0**	72.0	48.0	160.0	88.0	**78.0**	68.0	44.0	0.02
Creatinin clearance (mL/min per 1.73 m^2^)	L	242.2	137.1	**103.8**	79.5	16.91	158.1	93.3	**72.4**	54.6	15.2	<0.001
Glucosuria (negative = 0,+ = 1, ++ = 2, +++ = 3)	L	1.0	0.0	**0.0**	0.0	0.0	3.0	0.0	**0.0**	0.0	0.0	0.003
Hemoglobin (g/dL)	L	17.1	14.4	**13.6**	12.0	5.7	16.3	14.0	**12.6**	11.2	8.4	0.011
Monocytes (10^9^/L)	L	3.41	0.87	**0.47**	0.29	0.05	2.78	0.86	**0.59**	0.40	0.00	0.079
Blood sedimentation rate (mm/h)	L	105.0	27.5	**10.0**	4.0	2.0	114.0	31.5	**13.0**	6.0	1.0	0.086
Age (years)	H	88.0	67.75	**56.0**	46.25	18.0	92.0	76.0	**72.0**	66.75	39.0	<0.001
Smoking (packyears)	H	150.0	25.0	**1.0**	0.0	0	150.0	50.0	**15.0**	0.0	0.0	0.018
Number of children	H	7.0	2.0	**2.0**	1.0	0	6.0	3.0	**2.0**	1.0	0.0	0.028
Number of drugs (on admission)	H	10.0	4.0	**2.0**	1.0	0	14.0	8.0	**6.0**	4.0	0.0	<0.001
Number of medication (current)	H	11.0	5.0	**4.0**	2.0	0	14.0	9.0	**7.0**	5.0	2.0	<0.001
Number of admissions to this hospital	H	8.0	1.0	**0.0**	0.0	0	15.0	3.0	**1.0**	0.0	0.0	0.002
Number of risk factors (1-6)	H	6	3	**2**	1	0	6	4	**3**	3	0	<0.001
Disease activity score	O	1.83	1.22	**0.92**	0.67	0.25	2.67	1.79	**1.49**	1.27	0.41	<0.001

* Patients (7 without cardiovascular events, 6 with symptomatic atherosclerosis) who had incompressible ankle arteries ( = ABI>1.5) were excluded from this analysis.

# The numeric data obtained during the two study periods were compared using Mann-Whitney-U-Test.

§ Method by which the data was obtained: H = history, C = clinical examination, L = laboratory test, X = chest X-ray, E = electrocardiography, O = others.

### Statistical analysis

All statistical analyses were performed using SPSS version 12.0 (SPSS Inc., Chicago IL, USA). The numeric data obtained in the group of patients with symptomatic arteriosclerosis were compared to the patients without cardiovascular events using the Mann-Whitney U test. The presence or absence of cardiovascular risk factors was compared between the two groups using the χ^2^- test. Receiver operating curves (ROC) were used to assess the accuracy of the disease activity score as a diagnostic test. The disease activity score of a subgroup of 34 age- and sex-matched patients that were examined on two occasions at an interval of two years was compared using the Wilcoxon test. P-values<0.05 were supposed to indicate a significant difference between the groups. Unless indicated otherwise, median values are shown and interquartile range is given in brackets.

## Results

### Characteristics of the patient cohort

Of the 269 patients who participated in this study, 100 (37%) had symptomatic arteriosclerosis, i.e. they had suffered from cardiovascular events in the past. For 40 patients, the first cardiovascular event was the reason for the current admission to the hospital. For the other 60 patients, the first cardiovascular event did occur on average 5 [3]–[12] years ago. 110 (41%) had no history of cardiovascular events such as myocardial infarction, stroke, intermittent claudication, revascularization procedures or other disease defining conditions (Table 1). For 59 (22%) patients, the definite allocation to either one of these two groups was not possible. The characteristics of the patients without cardiovascular events in the past and of the patients with proven symptomatic arteriosclerosis are summarized in Table 1. On average, patients with symptomatic arteriosclerosis were older, and most conventional risk factors were significantly more common in this group. Smoking and a positive family history of cardiovascular events were the most prevalent risk factors in both patient groups (Table 1). Among vulnerable patients with symptomatic arteriosclerosis, 60% had coronary heart disease, 26% had cerebrovascular disease, 27% peripheral arterial occlusive disease, 7% aortic and 11% renal arteriosclerosis. For 27 patients, more than one vascular bed was affected by the disease. These rates of organ involvement by arteriosclerosis were similar to findings obtained in other population-based surveys [2], [6] and provide evidence for an unbiased patient selection.

The majority of the 25 variables that were found to be different between vulnerable patients and patients free of cardiovascular events was obtained in the bedside examination: 6 (24%) from the interview and 11 (44%) from the clinical exam. Only 5 (20%) were results from laboratory tests and 3 (12%) from chest X-ray or electrocardiogram (Table 2).

### Phentoypical description of vulnerable patients with arteriosclerosis using empiric clinical profiling

Most of the 25 variables which were significantly different in this systematic and comprehensive comparison of clinical data from symptomatic and asymptomatic patients reflect important and well-known clinical signs of arteriosclerosis or associated conditions: the anthropometric data reveal abdominal obesity [27], [28], the elevated systolic blood pressure [29] is caused by reduced wall compliance and the reduced ankle brachial index [23], [30] is a consequence of obstructed arteries. Cardiomegaly is a sign of left ventricular hypertrophy [31], QT prolongation may correlate with electric vulnerability [10], [11], diminished creatinin clearance and glucosuria indicate kidney injury [32], [33]. Anemia, monocytosis and elevated blood sedimentation rate are signs of chronic inflammation [34] and finally, the high number of drugs and repetitive hospitalizations are health economic aspects of symptomatic arteriosclerosis (Figure 1A). These categories, which emerged directly from the data analysis can be further used to manage arteriosclerosis both in individual patients and patient groups.

For example, the male patient whose data are shown in Figure 1A (white circles) and Figure 1B had a myocardial infarction three years ago. His disease profile draws the physician's attention to abdominal obesity as the major remaining, intuitively apparent sign of the disease under combined anti-hypertensive and lipid lowering treatment.

**Figure 2:**
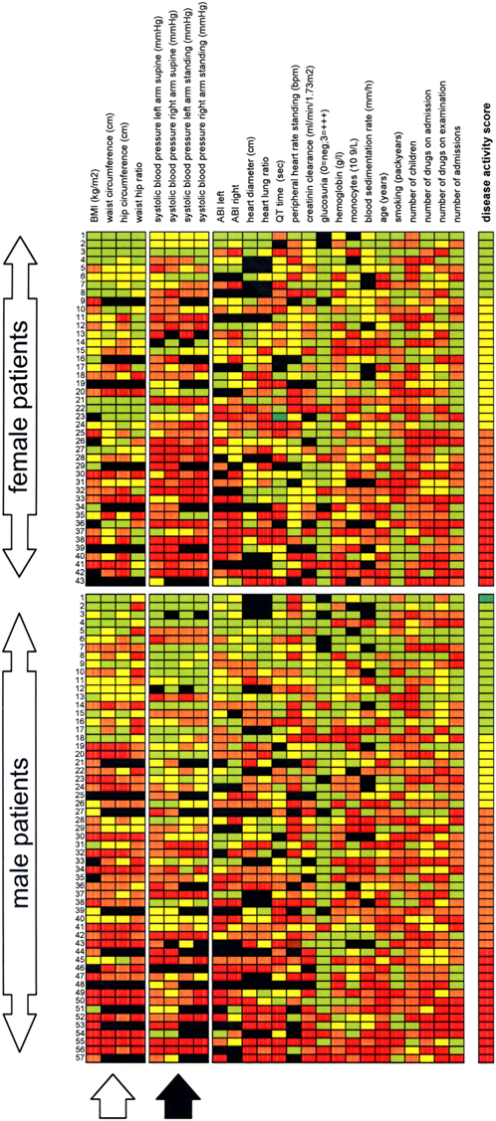
The clinical disease array. The color-coded empiric clinical disease profiles (see Figure 1B) of the 43 female and 57 male symptomatic patients were aligned and sorted according to disease activity score. Male and female patients were significantly different in anthropometric (white arrow) and blood pressure data (black arrow).

Since the rules of differential display were applied to analyze the clinical data, an alternative approach of empiric disease modeling was explored in this patient cohort. The color-coded profiles obtained from vulnerable patients were aligned in an array format (Figure 2). The cohort was split according to gender into female and male patients. This clinical array representation revealed two obvious, gender-specific differences in the phenotype of symptomatic arteriosclerosis. Female patients had higher, partially uncontrolled systolic blood pressure (145 (125–160) mmHg versus 130 (115–148) mmHg, P = 0.02, see Figure 2, black arrow) despite of taking the same number of antihypertensive drugs (on average 2 (1–2) drugs). In contrast, male patients were more obese having a significantly higher body mass index (27.7 (24.6–30.7) kg/m^2^ versus 25.4 (23.3–28.1) kg/m^2^, P = 0.04 and a higher waist hip ratio (1.02 (1.0–1.07) versus 0.91 (0.88–0.97), P<0.001) than female patients (Figure 2, white arrow). These gender-specific differences in anthropometric data and systolic blood pressure that were revealed by the empiric clinical profiling of arteriosclerosis in vulnerable patients were less pronounced (waist-hip ratio) or did follow the opposite direction (systolic blood pressure) in patients free of cardiovascular events.

### Diagnostic accuracy and prospective evolution of the disease activity score

**Figure 3:**
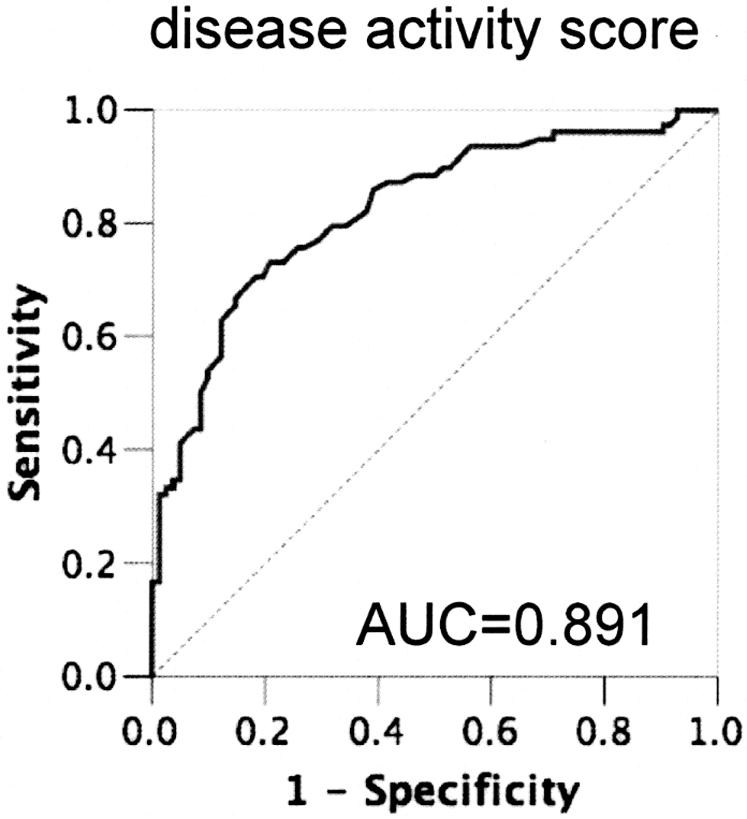
Diagnostic accuracy of the disease activity score. Sensitivity and specificity of the disease activity score to distinguish between asymptomatic and symptomatic patients is shown as receiver operating curve. Area under the curve is 0.891. The AUC for the null hypothesis is 0.5.

We evaluated the diagnostic accuracy of the disease activity score to identify individuals with symptomatic arteriosclerosis (Figure 3). The area under the curve (AUC) of the ROC was 0.891 (95% CI 0.799-0.983) and when a cut-off value of 1.05 was chosen, the sensitivity was 81% and the specificity 70% to diagnose systemic cardiovascular disease. For the number of risk factors, the AUC was 0.836 (95% CI 0.705-0.966), and for the 10-year risk according to the Framingham score, the AUC was 0.684 (95% CI 0.600-0.769). To add the number of risk factors to the disease activity score did not further improve the discriminating power of the test although the two variables showed only moderate correlation with each other (R = 0.48). In this group of patients, the disease activity score had the best diagnostic accuracy for symptomatic arteriosclerosis.

**Figure 4:**
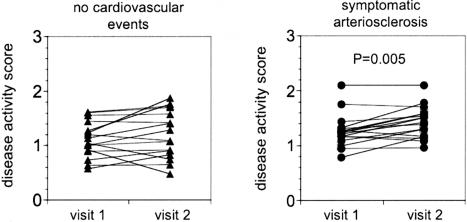
Evolution of the disease activity score with time. A selection of 34 age- and gender-matched patients (16 asymptomatic, 18 symptomatic, median age: 72 years) were reevaluated after 2 years. The disease activity score, assessed on visit 1 and visit 2 was compared using the Wilcoxon test.

The disease activity score increased with age. This age-dependent increase was much more pronounced in patients with symptomatic AS than in individuals free of cardiovascular events (Table 3). Therefore we tested the hypothesis that in patients with symptomatic arteriosclerosis the disease activity score deteriorated more rapidly with time than in asymptomatic individuals (Figure 4). 34 age and gender matched patients, 16 asymptomatic and 18 symptomatic (median age: 72 years), were reexamined after 2 years. During the two years of follow-up, one patient from the symptomatic group was re-admitted for a cardiovascular event (coronary angioplasty). In patients with symptomatic arteriosclerosis, the disease activity score deteriorated more rapidly (from 1.25 to 1.48, P = 0.005) than in asymptomatic individuals (from 1.09 to 1.19, not significant).

**Table 3:** Disease activity score in different age categories

	<40 years	40–59 years	60–79 years	>80 years
*Disease activity score*				
No cardiovascular events	0.8 (0.6–0.8)	0.8 (0.5–1.0)	1.0 (0.8–1.4)	1.0 (0.9–1.2)
	n = 17	n = 49	n = 38	n = 5
Symptomatic arteriosclerosis	0.6 (0.5–0.6)	1.0 (0.7–1.4)	1.4 (1.2–1.7)	1.7 (1.4–1.8)
	n = 2	n = 11	n = 72	n = 15
*P-value*	*0.352*	*0.106*	*<0.001*	*0.009*

## Discussion

We demonstrate that comprehensive clinical bedside examination including the interview, physical exam and a few paraclinical tests, can accurately describe the phenotype of patients with symptomatic arteriosclerosis. Comprehensive, quantitative phenotyping in the field of cardiovascular diseases was first discovered and developed by physiologists and formed the basis for sophisticated phenotype-genotype correlations in an animal model of hypertension [35]. For the clinical disease phenotype described herein we followed similar mathematical rules of data analysis. We found 25 numeric variables that contributed to the phenomenological description of patients with the disease, i.e. symptomatic arteriosclerosis characterized by cardiovascular events. The color coded, bar coded or quantitative percentile profiles serve to visualize the individual patient's disease phenotype. The clinical disease array that is formed by a cohort of vulnerable patients with symptomatic arteriosclerosis enables data analysis with the purpose to identify specific subgroups with associated conditions. For example, we found that in this group of patients, women with symptomatic arteriosclerosis had higher and partially uncontrolled systolic blood pressure despite of taking the same number of antihypertensive drugs as men. Finally, the calculated disease activity score had a respectable diagnostic power to discriminate between symptomatic and asymptomatic patients. It is at least as efficient as the number of cardiovascular risk factors. In addition, it deteriorated more rapidly over a short period of time in symptomatic patients. A finding that further supports its value as a biological activity marker of arteriosclerosis.

Based on the diagnostic accuracy of the disease activity score in this cohort of patients we assume that it may also represent a predictive test for patients admitted to this hospital. Both the absolute value of the disease activity score at any given time and its evolution with time could be important for the precise prediction of cardiovascular events in an individual patient. If it would reflect subclinical disease and identify patients susceptible for arterial injury and cardiovascular events it would clearly improve individualized long-term management of patients at risk. The present study has been performed in in-patients. Despite of this pre-selection bias the prevalence of cardiovascular events in the study population is similar to the reported data in outpatient cohorts [2], [6]. In order to improve the disease activity score as a predictive tool we will have to explore which variables change early in the course of arteriosclerosis, particularly in its asymptomatic stage. All these hypotheses and the deduced mathematical models have to be tested in prospective clinical trials and need to be confirmed in a population based cohort.

Finally, the individual empiric data-based disease profile could be used to test the efficacy of preventive or therapeutic interventions to treat arteriosclerosis. For example, any successful intervention leading to weight loss and reduced abdominal obesity may lead to quite obvious changes in a patient's phenotype and may even affect associated risk factors as shown by others [36]. The color coded disease profile may serve as a surrogate marker for the intuitive visualization of early responses to therapy.

Conditions that prevent or precipitate the development of symptomatic arteriosclerosis evolve with time and may also be different in various regions of the world [4], [9]. Therefore, this data-based, empiric clinical disease profile may differ in ten or twenty years from now and it may be different in medical centers in Asia, America or Africa. For the same reason, the reference range that defines this disease profile for symptomatic arteriosclerosis cannot be simply adopted by another institution. It should first be established on site. The quartile distribution of the different variables may represent a common ground for standardized comparisons of the disease phenotype and the activity score determined in different institutions. The optimal set of data, the size of the patient cohort and the time window for reference range calculations needs to be determined in future studies. Novel biomarkers will be tested for their capacity to improve the diagnostic accuracy of the clinical disease activity score presented herein.

In conclusion, affordable, available and accessible clinical data collected in a standardized manner and analyzed according to the rules of differential display result in an accurate description of the phenotype of patients with a complex disease, e.g. symptomatic arteriosclerosis. Data-based empiric clinical profiling visualizes an individual patient's disease phenotype quantitatively and it may form the basis of personalized risk assessments and interventions.

## Supporting Information

Table S1 Numerical variables obtained from the patients. Complete dataset from which the empiric clinical profile was obtained.
(0.54 MB DOC)
