# Supplementary material for: Individual Assessment of Arteriosclerosis by Empiric Clinical Profiling
Source: PLoS One. 2007 Nov 28;2(11):e1215. doi: 10.1371/journal.pone.0001215 (PMC2080761; doi:10.1371/journal.pone.0001215)
Supplement: Table S1 — Numerical variables obtained from the patients. Complete dataset from which the empiric clinical profile was obtained. (0.54 MB DOC) [file pone.0001215.s001.doc]

**Table S1: Numerical variables obtained from the patients.**

| **Code**  **number** | **Parameter** | **Method ^§^** | **No cardiovascular events**  **(total 110 patients)** | | **Symptomatic arteriosclerosis (total 100 patients)** | | **P-value ^#^** |
| --- | --- | --- | --- | --- | --- | --- | --- |
|  |  |  | Median | n (%) | Median | n (%) |  |
| I | Age (years) | H | 56.00 | 110 (100) | 72.00 | 100 (100) | <0.001 |
| II | Weight (kg) | C | 72.30 | 109 (99.1) | 75.35 | 98 (98) | 0.385 |
| III | Height (m) | C | 1.67 | 103 (93.6) | 1.68 | 89 (89) | 0.754 |
| IV | Body mass index (kg/m^2^) | C | 25.60 | 103 (93.6) | 26.40 | 88 (88) | 0.0085 |
| V | Waist circumference (cm) | C | 95.00 | 97 (88.2) | 99.50 | 84 (84) | 0.006 |
| VI | Hip circumference (cm) | C | 100.00 | 97 (88.2) | 101.50 | 84 (84) | 0.078 |
| VII | Waist hip ratio | C | 0.94 | 97 (88.2) | 1.00 | 84 (84) | 0.006 |
| VIII | Systolic blood pressure left arm supine (mmHg) | C | 130.00 | 108 (98.2) | 140.00 | 97 (97) | 0.001 |
| IX | Diastolic blood pressure left arm supine (mmHg) | C | 80.00 | 108 (98.2) | 80.00 | 97 (97) | 0.421 |
| X | Systolic blood pressure right arm supine (mmHg) | C | 130.00 | 109 (99.1) | 135.00 | 95 (95) | 0.005 |
| XI | Diastolic blood pressure right arm supine (mmHg) | C | 80.00 | 109 (99.1) | 80.00 | 95 (95) | 0.717 |
| XII | Peripheral heart rate  supine (bpm) | C | 76.00 | 109 (99.1) | 72.00 | 100 (100) | 0.124 |
| XIII | Systolic blood pressure left arm standing (mmHg) | C | 120.00 | 101 (91.8) | 130.00 | 79 (79) | <0.001 |
| XIV | Diastolic blood pressure left arm standing (mmHg) | C | 80.00 | 101 (91.8) | 80.00 | 79 (79) | 0.464 |
| XV | Systolic blood pressure right arm standing (mmHg) | C | 120.00 | 100 (90.9) | 130.00 | 78 (78) | 0.007 |
| XVI | Diastolic blood pressure right arm standing (mmHg) | C | 80.00 | 101 (91.8) | 80.00 | 78 (78) | 0.761 |
| XVII | Peripheral heart rate  standing (bpm) | C | 80.00 | 102 (92.7) | 78.00 | 82 (82) | 0.02 |
| XVIII | Central heart rate  supine (bpm) | C | 76.00 | 109 (99.1) | 72.00 | 100 (100) | 0.311 |
| XIX | Ankle brachial index right * | C | 1.14 | 95 (86.4) | 1.00 | 80 (80) | <0.001 |
| XX | Ankle brachial index left * | C | 1.15 | 86 (78.2) | 1.02 | 78 (78) | <0.001 |
| XXI | Hemoglobin (g/dL) | L | 13.60 | 110 (100) | 12.55 | 100 (100) | 0.011 |
| XXII | Leukocytes (10 9/L) | L | 8.50 | 109 (99.1) | 8.30 | 100 (100) | 0.896 |
| XXIII | Thrombocytes (10 9/L) | L | 253.00 | 109 (99.1) | 259.00 | 100 (100) | 0.738 |
| XXIV | Neutrophile granulocytes  (10 9/L) | L | 5.61 | 94 (85.5) | 5.66 | 90 (90) | 0.991 |
| XXV | Eosinophilic granulocytes  (10 9/L) | L | 0.07 | 96 (87.3) | 0.12 | 91 (91) | 0.077 |
| XXVI | Basophilic granulocytes  (10 9/L) | L | 0.04 | 96 (87.3) | 0.03 | 90 (90) | 0.756 |
| XXVII | Lymphocytes (10 9/L) | L | 1.70 | 96 (87.3) | 1.60 | 91 (91) | 0.256 |
| XXVIII | Monocytes (10 9/L) | L | 0.47 | 96 (87.3) | 0.59 | 89 (89) | 0.079 |
| XXIX | HbA1c (%) | L | 6.10 | 18 (16.4) | 7.10 | 33 (33) |  |
| XXX | Blood glucose level after overnight fast (mmol/L) | L | 6.20 | 79 (71.8) | 6.00 | 73 (73) |  |
| XXXI | Cholesterol total (mmol/L) | L | 5.00 | 45 (41) | 4.60 | 68 (68) |  |
| XXXII | LDL cholesterol (mmol/L) | L | 3.28 | 42 (38.2) | 2.65 | 62 (63) |  |
| XXXIII | HDL cholesterol (mmol/L) | L | 1.08 | 44 (40) | 1.05 | 67 (67) |  |
| XXXIV | Triglycerides (mmol/L) | L | 1.10 | 43 (39.1) | 1.60 | 60 (60) |  |
| XXXV | Kreatinin (µmol/L | L | 62.00 | 107 (97.3) | 77.00 | 100 (100) | <0.001 |
| XXXVI | Body surface area (m^2^) | L | 1.84 | 103 (93.6) | 1.87 | 89 (89) | 0.577 |
| XXXVII | Kreatinin clearance  (mL/min per 1.73 m^2^) | L | 103.80 | 100 (90.9) | 72.40 | 89 (89) | <0.001 |
| XXXVIII | Blood sedimentation rate (mm/h) | L | 10.00 | 79 (71.8) | 13.00 | 80 (80) | 0.086 |
| XXXIX | C-reactive protein (mg/L) | L | 7.00 | 104 (94.6) | 9.00 | 97 (97) | 0.534 |
| XL | Creatine kinase (U/L) | L | 98.50 | 84 (76.4) | 107.00 | 86 (86) | 0.217 |
| XLI | Creatin kinase, MB form (U/L) | L | 4.00 | 25 (22.7) | 6.00 | 52 (52) |  |
| XLII | Troponin I (ng/mL) | L | 0.02 | 26 (23.6) | 0.04 | 43 (43) |  |
| XLIII | GOT/ASAT (U/L) | L | 23.00 | 104 (94.6) | 25.00 | 95 (95) | 0.272 |
| XLIV | GPT/ALAT (U/L) | L | 24.00 | 104 (94.6) | 23.00 | 95 (95) | 0.903 |
| XLV | Alkaline phosphatase (U/L) | L | 67.00 | 99 (90) | 77.00 | 88 (88) | 0.197 |
| XLVI | Gamma glutamyl transpeptidase GGT (U/L) | L | 41.00 | 43 (39.1) | 61.50 | 34 (34) |  |
| XLVII | D-dimer (μg/L) | L | 1.03 | 23 (20.9) | 2.02 | 11 (11) |  |
| XLVIII | Brain natriuretic peptide (pg/mL) | L | 256.50 | 6 (5.5) | 220.00 | 17 (17) |  |
| XLIX | Thyroidea stimulating hormone (mU/L) | L | 1.12 | 36 (32.7) | 1.47 | 41 (41) |  |
| L | INR | L | 1.10 | 107 (97.3) | 1.10 | 92 (92) | 0.316 |
| LI | Proteinuria  (negative=0,+=1, ++=2, +++=3) | L | 0.00 | 110 (100) | 0.00 | 100 (100) | 0.23 |
| LII | Glucosuria  (negative=0,+=1, ++=2, +++=3) | L | 0.00 | 110 (100) | 0.00 | 100 (100) | 0.003 |
| LIII | Urine leukocytosis (negative=0,+=1, ++=2, +++=3) | L | 0.00 | 110 (100) | 0.00 | 100 (100) | 0.647 |
| LIV | Heart rate in ECG (bpm) | E | 77.00 | 98 (89.1) | 73.00 | 93 (93) | 0.187 |
| LV | QT time (sec) | E | 0.37 | 95 (86.4) | 0.40 | 90 (90) | <0.001 |
| LVI | Sokolov index right  ventricle (mV) | E | 0.30 | 96 (87.3) | 0.40 | 92 (92) | 0.494 |
| LVII | Sokolov index left  ventricle (mV) | E | 1.70 | 97 (88.2) | 1.60 | 92 (92) | 0.514 |
| LVIII | Maximal exercise capacity stress test (W) | O | 137.50 | 10 (9.1) | 100.00 | 9 (9) |  |
| LIX | Lung diameter (cm) | X | 28.55 | 70 (63.6) | 29.60 | 77 (77) | 0.35 |
| LX | Heart diameter (cm) | X | 13.50 | 70 (63.6) | 15.60 | 77 (77) | <0.001 |
| LXI | Heart lung ratio | X | 0.47 | 70 (63.6) | 0.51 | 77 (77) | <0.001 |
| LXII | Left ventricular ejection fraction (%) | O | 40.50 | 14 (12.7) | 40.00 | 37 (37) |  |
| LXIII | Age father at time of death (years) | H | 73.00 | 71 (64.6) | 74.00 | 78 (78) | 0.09 |
| LXIV | Age mother at time of death (years) | H | 80.00 | 54 (49.1) | 80.00 | 76 (76) | 0.88 |
| LXV | Number of siblings | H | 2.00 | 87 (88.2) | 2.00 | 86 (86) | 0.693 |
| LXVI | Number of children | H | 2.00 | 110 (100) | 2.00 | 100 (100) | 0.028 |
| LXVII | Number of drugs (on admission) | H | 2.00 | 110 (100) | 6.00 | 100 (100) | <0.001 |
| LXVIII | Number of drugs for the treatment of atherosclerosis (on admission) | H | 0.00 | 110 (100) | 3.50 | 100 (100) | <0.001 |
| LXIX | Number of drugs (current) | O | 4.00 | 110 (100) | 7.00 | 100 (100) | <0.001 |
| LXX | Number of drugs for the treatment of athersclerosis (current) | O | 0.00 | 110 (100) | 4.00 | 100 (100) | <0.001 |
| LXXI | Number of admissions to this hospital | H | 0.00 | 108 (98.2) | 1.00 | 96 (96) | 0.002 |
| LXXII | Packyears | H | 1.00 | 110 (100) | 15.00 | 100 (100) | 0.018 |
| LXXIII | Number of standard alcoholic drinks | H | 0.00 | 110 (100) | 0.00 | 100 (100) | 0.127 |
| LXXIV | If female: age at menarch (years) | H | 14.00 | 55 (91.7 of female patients) | 13.25 | 40 (93.0 of female patients) | 0.873 |
| LXXV | If female: age at menopause (years) | H | 48.00 | 29 (48.3 of female patients) | 50.00 | 35 (81.4 of female patients) | 0.354 |
|  |  |  |  |  |  |  |  |
| LXXVI | Number of cardiovascular risk factors | H | 2.00 | 110 (100) | 3.00 | 100 (100) | <0.001 |
| LXXVII | Disease activity score | O | 0.92 | 110 (100) | 1.51 | 100 (100) | <0.001 |

^#^ The data of the patient groups are shown as median values. The two patient groups were compared by Mann-Whitney-U-Test. Data sets with P<0.1were selected for the disease activity score.

* Patients (7 without cardiovascular events, 6 with symptomatic arteriosclerosis) who had incompressible ankle arteries (=ABI>1.5) were excluded from this analysis.

^§^ Method by which the data was obtained: H=history/interview, C=clinical examination, L=laboratory test, X=chest X-ray, E=electrocardiography, O=others.
